# MiRNAs, Myostatin, and Muscle MRI Imaging as Biomarkers of Clinical Features in Becker Muscular Dystrophy

**DOI:** 10.3390/diagnostics10090713

**Published:** 2020-09-18

**Authors:** Roberta Marozzo, Valentina Pegoraro, Corrado Angelini

**Affiliations:** IRCCS San Camillo Hospital, 30126 Venice, Italy; roberta.marozzo@ospedalesancamillo.net (R.M.); Valentina.pegoraro@ospedalesancamillo.net (V.P.)

**Keywords:** Becker muscular dystrophy, follistatin, microRNAs, muscle MRI, myostatin

## Abstract

Becker muscular dystrophy (BMD) is an X-linked recessive disorder caused by dystrophin gene mutations. The phenotype and evolution of this muscle disorder are extremely clinical variable. In the last years, circulating biomarkers have acquired remarkable importance in their use as noninvasive biological indicators of prognosis and in monitoring muscle disease progression, especially when associated to muscle MRI imaging. We investigated the levels of circulating microRNAs (myo-miRNAs and inflammatory miRNAs) and of the proteins follistatin (FSTN) and myostatin (GDF-8) and compared results with clinical and radiological imaging data. In eight BMD patients, including two cases with evolving lower extremity weakness treated with deflazacort, we evaluated the expression level of 4 myo-miRNAs (miR-1, miR-206, miR-133a, and miR-133b), 3 inflammatory miRNAs (miR-146b, miR-155, and miR-221), FSTN, and GDF-8 proteins. In the two treated cases, there was pronounced posterior thigh and leg fibrofatty replacement assessed by muscle MRI by Mercuri score. The muscle-specific miR-206 was increased in all patients, and inflammatory miR-221 and miR-146b were variably elevated. A significant difference in myostatin expression was observed between steroid-treated and untreated patients. This study suggests that microRNAs and myostatin protein levels could be used to better understand the progression and management of the disease.

## 1. Introduction

Becker muscular dystrophy (BMD) is an X-linked recessive disorder and has an incidence of 1 in 18,518 male births [1] and a prevalence of 0.01 in South Africa, 0.1 to 0.2 in Asia, 0.1 to 0.7 (per 10,000 males) in European countries [2]. It is caused by mutations of the DMD gene that encodes the muscle-specific dystrophin protein. The pivotal function of dystrophin is to link the cytoskeletal actin and dystrophin-associated protein complex to the extracellular matrix to stabilize the sarcolemma [3]. Some mutations of dystrophin compromise its binding at the sarcolemma and cause a specific sequence of muscle abnormalities such as muscle necrosis, altered mitochondrial metabolism, disruption of the sarcolemma, abnormality of calcium homeostasis, and reactive macrophagic inflammation [4]. The BMD typical clinical features consist of proximal muscle wasting and weakness, high creatine kinase (CK), cramps, myalgia, myoglobinuria, mild myopathy, and cardiomyopathy with fibrosis [5]. In young adults, the proximal muscle wasting and weakness are more evident at the thigh and pelvic girdle muscles and pronounced calf hypertrophy might be present [6]. At the mild end of the BMD spectrum are patients characterized by muscle hypertrophy of the calves, cramps, and elevated CK levels, but virtually no muscle wasting or weakness [7,8]. Becker muscular dystrophy differential diagnosis is important to distinguish it from other myopathies with muscle weakness; BMD patients remain ambulatory after the age of 16 years while Duchenne muscular dystrophy (DMD) patients lose ambulation and become wheelchair-bound by age of 12 [7,9]. BMD has a later onset and is clinically less severe compared to DMD. At the molecular level. DMD gene mutations disrupt the translational reading frame, which results in complete loss of dystrophin. In BMD, the mutation maintain the reading frame and result in truncated dystrophin protein with abnormal function [7,10]. In some instances, BMD could be confused with polymyositis, an idiopathic inflammatory myopathy characterized by bilateral proximal muscle weakness. BMD can be distinguished for the presence of calf pseudohypertrophy [7,11] and frequent cardiac involvement [12].

Another muscle dystrophy group difficult to differentiate from BMD is limb-girdle muscular dystrophy, the hallmark of which is the absence of overt calf muscle pseudohypertrophy [13,14].

No definitive cure for BMD has been identified; however, steroid therapy appears useful to improve muscle function, to slow the decline of muscle strength, and to prolong in dystrophinopathy walking ability [5,15]. In recent years, a potential strategy to detect and follow dystrophic symptoms could be the use of noninvasive biomarkers to monitor the disease.

MicroRNAs (miRNAs) are a class of small non-coding RNAs molecules with 22 nucleotides in length that regulate gene expression at the post-transcriptional level by destabilizing the mRNA and translation silencing [16]. MiRNAs are involved in several pathological and physiological processes such as development, proliferation, differentiation, and cell death. MiRNAs are expressed in muscle and stable in biofluids, such as plasma, serum, and urine [17]. Altered levels of circulating miRNAs have been linked to several neuromuscular diseases [18].

A specific group of circulating miRNAs is muscle tissue specific and they are called “myo-miRNAs” (miR-1, miR-206 and miR-133). MiR-1 and miR-133 are expressed in cardiac and skeletal muscle and are involved in the proliferation and differentiation processes [19]; miR-206 is a skeletal muscle-specific miRNA expressed in satellite cells and involved in muscle development and regeneration [20]. Several studies demonstrated that the serum levels of myo-miRs are increased in DMD and BMD patients [21]. Another common feature of muscular dystrophy is the inflammatory response that triggers a cascade of inflammatory cytokines and subsequently miRNAs induction. The activation of the inflammatory system is due to the muscle injury and the subsequent macrophagic reaction, which occurs to repair the muscle damage and to promote the regeneration process. Three inflammatory miRNAs (miR-146b, miR-221 and miR-155) were found to be dysregulated in several muscular dystrophies [22].

Myostatin (Mstn), also named “GDF-8,” is a member of the transforming growth factor β (TGFβ) superfamily and acts as a negative regulator of skeletal muscle growth [23]. It is known that the myostatin antagonist is follistatin (FST), a glycosylated secreted protein that controls muscle mass through different pathways [24].

We examined the serum levels of myo-miRNAs (miR-1, miR-206 and miR-133), inflammatory miRNAs (miR-146b, miR-155 and miR-221), and plasma follistatin and myostatin in eight long-standing monitored BMD patients, two of which were treated with deflazacort. We tried, also, to establish a correlation between the clinical changes observed by MRI and the selected circulating biomarkers.

## 2. Materials and Methods

We enrolled 8 BMD patients (aged 14–46 years) diagnosed by previous identification of mutations in the dystrophin gene. Genetic DNA study for dystrophin deletions was done in collaboration with TIGEM or other Genetic Units. Diagnosis of BMD was done in muscle biopsy and quantity and molecular weight (M.W.) of dystrophin were assessed by Western blotting as previously described [8].

Inclusion criteria of our patients were: genetically proven BMD with preserved walking ability and no contraindications to perform muscle MRI.

In all patients, the ability to walk was preserved. Two of them were treated with deflazacort in alternate day regime for several years and were followed for a period of over 15 years after diagnosis. A control group was formed by 6 healthy male subjects age-matched to patients, they all presented normal CK (range 22–198 U/L).

At Neuromuscular Center, Department of Neurobiology Lab at IRCCS San Camillo, the neuromuscular examination and blood collection were done at the time of MRI exam, and previous exercise activity was monitored. BMD patients and controls underwent peripheral blood venous puncture, their serum and plasma were collected and stored at −80 °C in the Biobank of the Neuromuscular Center, IRCCS San Camillo, until use. All patients (or parents for children under 18 years) and healthy individuals signed the informed consent to participate to the study specific for the biological samples collection, muscle MRI, and clinical data recording, during the medical visit. The approval (10 January 2017) from the local Ethics Committee was obtained for this study.

### 2.1. MicroRNAs Method

Total RNA was extracted from 400 μL of serum samples using the miRNeasy Mini Kit (Qiagen, Hilden, Germany), as recommended by the manufacturer. Briefly, 5 µL of total RNA of each sample was first subjected to a reverse transcription reaction to obtain cDNAs, subsequently, real-time polymerase chain reaction (qRT-PCR) was performed using the CFX96™ Real-Time PCR Detection System (Biorad, Hercules, CA, USA), along with specific TaqMan MicroRNA Assay (Thermo Fisher Scientific, Waltham, MA, USA) (Table S1). The expression level of each miRNA was normalized to average levels of miR-16, U6 snRNA, and miR-39-3p of *Caenorhabditis elegans,* the first two were used as endogenous controls, while the last as spike-in miRNA as previously described [25]. Data were obtained by triplicate experiments. The comparative 2^−ΔΔ*C*t^ method was used for the miRNA relative expression. 

### 2.2. Myostatin/Follistatin Method

The plasma concentration of myostatin and follistatin proteins were measured using specific ELISA immunoassay kits (myostatin: KR1012, Immunodiagnostic, Bens-Heim, Germany) (follistatin: ab113319, Abcam, Cambridge, MA, USA) according to the manufacturer’s protocol. The experiment was performed in duplicate. For myostatin analysis, 20 μL of each plasma was diluted 1:10 in sample buffer solution; for follistatin assay, 25 μL of plasma were diluted 1:10 in buffer solution. 

The absorption was read at 450 nm against 620 nm as reference using the Infinite F50 Tecan microplate reader for both analyses. To calculate the myostatin concentration, the Magellan program 4-parameter algorithm has been used, whereas the follistatin levels were determined drawing the best-fit straight line through the standard points as previously described [26].

### 2.3. Statistical Analysis

Statistical analysis was carried out using R-Studio, and the Wilcoxon–Mann–Whitney test was used for small samples. *p*-value of ≤0.05 was considered statistically significant.

### 2.4. Muscle MRI Method

The skeletal muscles of both lower limbs of patients have been evaluated using magnetic resonance imaging (MRI) on 1.5-Tesla Philips Apparatus using a T1-weighted MRI system. One patient refused to undergo the exam because of anxiety. The fibrofatty replacement of muscle observed by muscle MRI was evaluated on T1 sequences, using Mercuri score, which classifies in 6 stages the extent of muscle degeneration [27]. We evaluated the following muscles: quadriceps femoris (rectus femoris, vastus medialis, and vastus lateralis), muscle hamstrings (biceps femoris, semimembranosus, and semitendinosus), and muscles in the thigh and in the leg (tibialis anterior and gastrocnemius).

The evaluation of the Mercuri score was done independently by two distinct operators (R.M. and V.P.).

## 3. Results

The clinical data of each patient are summarized in Table 1.

### 3.1. Expression Levels of myo-miRNAs

**Figure 1:**
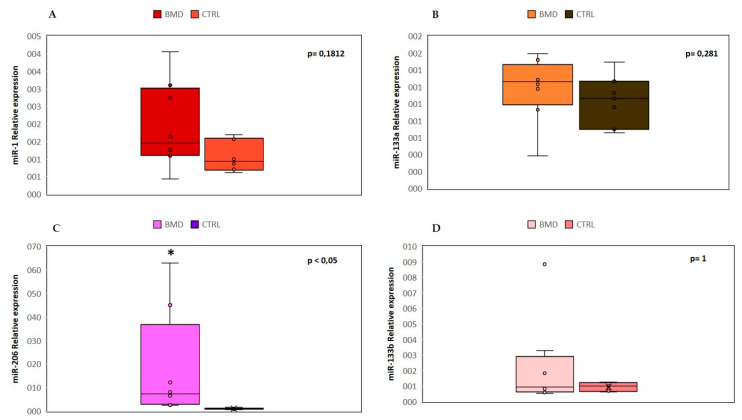
Comparison of the serum levels of myo-miRNAs in eight Becker muscular dystrophy (BMD) patients and six control subjects. Box plots show relative expression of miR-1 (**A**), miR-133a (**B**), miR-206 (**C**), and miR-133b (**D**) determined by quantitative real-time polymerase chain reaction (qRT-PCR). Single points show the individual myo-miRNAs levels, the box represents the quartiles (first and third) divided into two parts by the median (second quartile), and the maximum and minimum values are also indicated. The asterisk indicates significant *p*-values (*p* ≤ 0.05) between patients and controls using the Wilcoxon–Mann–Whitney test. BMD: Becker muscular dystrophy. CTRL: controls.

We analyzed the expression levels of four “canonical” myo-miRNAs (miR-1, miR-133a, miR-133b and miR-206) in the serum of 8 BMD patients by real-time PCR, as shown in Figure 1.

We found a significant (*p* < 0.05) upregulation of miR-206 in all patients, and in two cases (patient 1 and patient 8), there was an over 45-fold increase as compared to controls. No significant differences were observed in the mean value of miR-1, miR-133a, and miR-133b, but patient 8 showed a 9-fold higher value of miR-133b than control mean. The high variability detected in patient 8 could be attributed to a strenuous and eccentric muscle exercise done, few days before serum sample collection.

### 3.2. Expression Levels of Inflammatory MiRNAs

**Figure 2:**
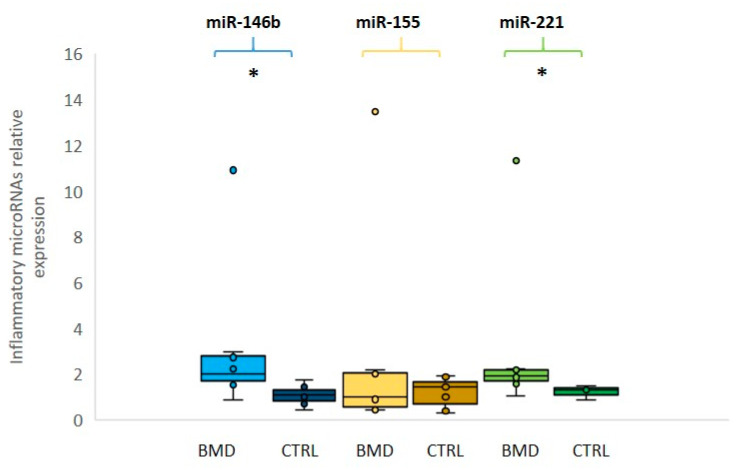
Relative expression of inflammatory microRNAs in BMD patients. Box plots showing the levels of miR-146b (light blue for patients and dark blue for controls), miR-155 (yellow for patients and dark yellow for controls) and miR-221 (green for patients and dark green for controls) (by qRT-PCR analyses in the serum of eight BMD patients and in six healthy controls). Data were significant, *p*-values (*p* ≤ 0.05), for miR-221 and miR-146b between patients and controls. Single points show the individual inflammatory miRNAs levels, the box represents the quartiles (first and third) divided into two parts by the median (second quartile), and the maximum and minimum values are also indicated BMD: Becker muscular dystrophy; CTRL: controls; * indicates significance (*p* ≤ 0.05).

We then evaluated the expression levels of three selected inflammatory miRNAs (miR-146b, miR-155, and miR-221): miR-146b and miR-221 were found to be significantly overexpressed (*p* ≤ 0.05) in BMD patients in comparison to the control group, whereas miR-155 was not significantly different between patients and controls (Figure 2). In this group of inflammatory miRNAs, patient 8 showed over 10-fold increased levels as compared to control mean, in line with damage during the previous eccentric exercise and subsequent inflammatory macrophage reaction.

### 3.3. Quantitative Expression of Myostatin/Follistatin

**Figure 3:**
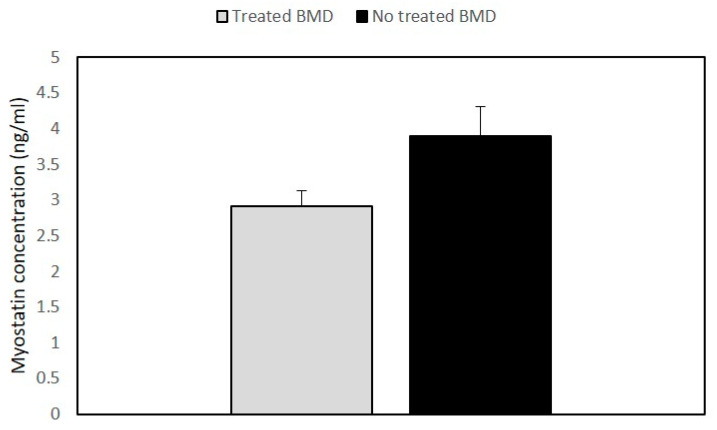
Histogram showing the concentration of myostatin in BMD patients. Quantitative myostatin expression was compared between treated (*n* = 2) and untreated BMD (*n* = 6) patients using the ELISA test. Data are expressed as mean + standard deviation.

The analysis of the myostatin (GDF-8) and follistatin (FST) concentrations was an important aim of our study. The results showed that GDF8 and FST levels were not significantly different in BMD patients as compared to the control group (Data shown in Supplementary Figure S1). However, when we compared myostatin levels between the treated and the untreated patients’ groups, the steroid-treated BMD group showed lower values of GDF8 than untreated patients (Figure 3).

### 3.4. Muscle MRI Changes

Mercuri score of patient’s lower limbs is reported in Table S2. 

**Figure 4:**
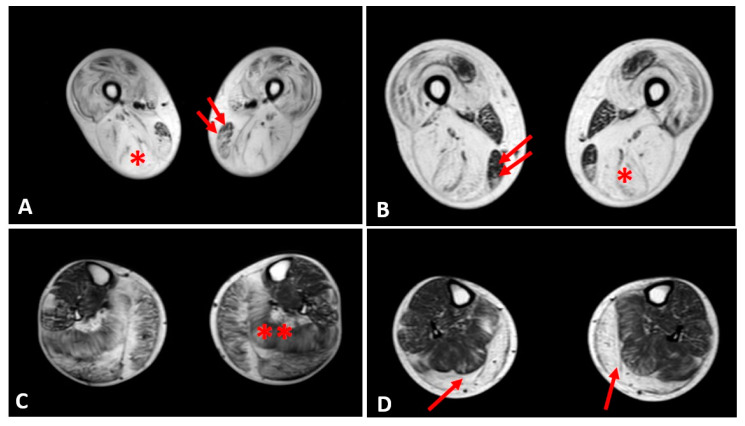
Evaluation of fibrofatty infiltration in lower limbs in two steroid-treated BMD patients. T1-sequences of thigh muscles shows marked atrophy of quadriceps and hamstrings (single asterisk) in both patients sparing of gracilis (double arrows) and sartorius muscles (**A**,**B**). In leg muscles, gastrocnemius and soleus muscles (double asterisk) presented advanced fatty and connective tissue infiltration in patient 1 (**C**), whereas marked atrophy and moderate fatty infiltration were observed in gastrocnemius muscles (single arrow) of patient 2 (**D**).

Thigh muscles showed significant fatty infiltration of muscles in the anterior and posterior compartment in patient 1 and patient 2 who were treated with steroids. Both quadriceps and posterior hamstrings muscles were equally affected; only gracilis and sartorius muscles were spared in these two patients (Figure 4).

In the other patients, the thigh muscles were normal or slightly compromised, except for patients 7 and 8, in whom the fatty tissue infiltration in hamstring muscles reached Mercuri score 2a. 

The posterior leg compartment was mostly affected in the two patients treated by steroids, showing severe fatty infiltration in gastrocnemius muscle. In the anterior leg, tibialis anterior was slightly involved; these 2 patients showed a Mercuri score 2a (Figure 4).

The other patients did not present a specific pattern of leg muscles involvement.

## 4. Discussion

Currently, a few studies that report consistently and effective biomarkers for BMD are present in the literature compared to DMD data. Our study is directed to add new results of circulating biomarkers in the serum and plasma of BMD patients. 

Several studies showed that the levels of myo-miRNAs in the serum of patients with DMD were altered in correlation with the progression of the dystrophic pathology [28] when there is consistent damage of muscle and in the presence of a steroid regimen treatment that delayed muscle deterioration [29,30]. Upregulation of the myo-miRNAs miR-1, miR-133a, miR-133b, and miR-206 has been previously reported in serum from DMD patients [28,31]; in particular, miR-206 levels were correlated with the different diagnosis of DMD and BMD [32]. Our data provide evidence that miR-206 was significantly upregulated in all BMD patients, whereas the expression levels of miR-1 and miR-133a/b was variable among BMD patients. Our results strongly support the observations that miR-206 is higher in BMD patients, and we did not observe difference in the levels of miR-206 between treated and untreated patients.

The investigation of miRNAs involved in the inflammation process showed a significant increase of miR-146b and miR-221 levels in BMD patients, whereas no significant differences have been found in miR-155 compared to the controls. These data could be explained by the observation that our patients present a relatively severe and chronic form of muscle disease (due to a fairly stable condition of muscle degeneration), whereas miR-155 acts during the acute process of the disease [33] since it has been found to regulate the myogenic regeneration process in mdx mouse model of DMD following injury by the control of the balance between pro-inflammatory and anti-inflammatory macrophages [34]. An important function of glucocorticoids is to relieve inflammation, and it has been shown that this process occurs through the suppression of miR-155 [35].

MiR-146b and miR-221 seem to have an opposite role of miR-155, as they are activated during chronic inflammatory conditions. In particular, miR-146b suppresses the production of inflammatory factors and its overexpression counteracts the activation of NFkB [36,37], whereas miR-221 is involved in the macrophagic phase [38]. MiR-146b is also called dystrophin-targeting miRNAs (DTM) because it downregulates dystrophin by binding to its 3′ untranslated region (UTR) and it is induced by inflammation [39]. In DMD muscle, Fiorillo et al. [39] demonstrated that miR-146b was upregulated when the dystrophin amount decreased in muscle fibers. In the Eisenberg study [22], miR-146b was significantly upregulated in the muscle of BMD and DMD patients and this is consistent with our results obtained in serum.

Taken together, our data might help to explain the role of circulating miRNAs in BMD, even if they are derived from a limited series of patients.

One original result from our study is derived from the correlation between miRNAs levels and the degree of fibrofatty replacement of muscles using muscle MRI imaging and the Mercuri score of impairment. In several muscle disorders, the changes observed by Mercuri score and functional muscle performances present a linear correlation [40,41]. In our study, the major muscle groups that presented the highest Mercuri score (hamstrings, quadriceps, and gastrocnemius) were found in the two patients treated with steroids. This could be explained by the observation that patient 1 and patient 2 were treated when they presented an advanced clinical stage of the disease, characterized by waddling gait and muscle function deterioration, as compared to the others, who were only mildly affected. In these two patients, the long-lasting steroid treatment might have helped them to prolong walking ability and to relatively preserve their muscle strength; however, their disease course and whole muscle impairment appear to be much more severe than in the other patients.

In addition, we found that the two steroid-treated-patients presented low levels of myostatin compared to the other BMD patients and normal concentration of follistatin protein. This result shows that the balance between myostatin and follistatin was compromised. Circulating myostatin, which is mainly produced by muscle tissue, has been considered as a biomarker of atrophy and muscle wasting and is associated with disease progression [42,43]. Our data support the observation that the two dystrophinopathy patients who presented advanced muscle atrophy are the only cases showing reduced myostatin levels, that correlated with their reduced muscle mass.

Concerning follistatin, the pivotal function of this protein is the inhibition of the myostatin pathway in order to regulate the muscle mass in muscular dystrophies. Several myostatin inhibitory drugs have been evaluated in neuromuscular diseases, but so far, the clinical results are limited [44]. In a gene therapy trial based on the use of adeno-associated virus (AAV) vector with preliminary prednisone treatment, follistatin was injected in six BMD ambulatory patients, and there was in four patients increase in the distance walked in six-minute test (6MWT) and reduced endomysial fibrosis, muscle hypertrophy especially at high dose [45,46]. It is possible that such follistatin gene therapy combined with long-term deflazacort regime could be used in the future to treat the symptoms and maintain muscle strength in BMD patients. In future studies, sonography could be also utilized as alternative to muscle MRI, to evaluate quality of muscle mass since this technique is a cost-effective tool recently developed for evaluation of metabolic syndrome and adults dynapenia [47,48]. We propose that circulating miRNAs and myostatin could be used as a noninvasive prognostic biomarkers to monitor the progression and the treatment of the disease. We suggest as a clinical prospective and possible applicability of this study the combined use of circulating biomarkers with muscle MRI and sonography in order to improve diagnosis and disease management in both young dystrophinopathy and chronic adult myopathy patients.

## Figures and Tables

**Table 1:** Clinical features and dystrophin gene mutation in Becker muscular dystrophy (BMD) patients.

	Age at Study (Years)	Age at Onset (Years)	Deletion in the Dystrophin Gene	Dystrophin Quantity (%) and M.W. (kDa) *	Creatine Kinase Levels (U/L)	Muscle Involvement	Left Ventricular Ejection Fraction **	Treatment and Duration
Patient 1	42	5	Exons 45–47	35%, 370 kDa	1202	HyperCKemia, waddling gait, calf hypertrophy, quadriceps weakness	40–55%	Deflazacort ACE inhibitors 26 years
Patient 2	40	17	Exons 45–49	60%, 380 kDa	452	HyperCKemia, pes cavus, difficulty rising from the floor, calf atrophy, weakness	55%	Deflazacort 20 years
Patient 3	33	1 1/2	Exons 48–51	90%, 370 kDa	189	Slight hyperCKemia, muscle cramps, quadriceps weakness	65%	No
Patient 4	41	6	Exons 31–44	35%, 320 kDa	3086	HyperCKemia, mild mitral valve insufficiency	55%	No
Patient 5	17	6	Exons 45–47	20%, 380 kDa	1092	HyperCKemia, calf hypertrophy	64%	No
Patient 6	14	3	Exons 45–51	50%, 370 kDa	668	HyperCKemia, mild winging scapulae	65%	No
Patient 7	30	1 1/2	Exons 48–49	50%, 380 kDa	1305	HyperCKemia, slight scoliosis, calf hypertrophy	55%	No
Patient 8	45	Childhood	Exons 47–49	N.A.	597–943	HyperCKemia, slight scapular winging, calf hypertrophy	55%	No

N.A.: not available. *: dystrophin quantity was expressed as percentage of control mean; dystrophin molecular weight (M.W.) was measured by comparison with those on normal muscle (400 kDa) as determined by Western blotting on muscle biopsy tissue obtained at the time at diagnosis. **: normal values 53–63%.

## Supplementary Materials

The following are available online at https://www.mdpi.com/2075-4418/10/9/713/s1, Table S1: TaqMan microRNA primer sequences. Table S2: Fatty infiltration with Mercuri score in lower limb muscles. Figure S1: Myostatin (Myo) and follistatin (Foll) levels in plasma of eight Becker muscular dystrophy (BMD) and six controls. Data were presented as mean + standard deviation.
