# Supplementary material for: MiRNAs, Myostatin, and Muscle MRI Imaging as Biomarkers of Clinical Features in Becker Muscular Dystrophy
Source: Diagnostics (Basel). 2020 Sep 18;10(9):713. doi: 10.3390/diagnostics10090713 (PMC7554733; doi:10.3390/diagnostics10090713)
Supplement: Supplementary file 1 [file diagnostics-10-00713-s001.pdf]

Supplementary Materials:

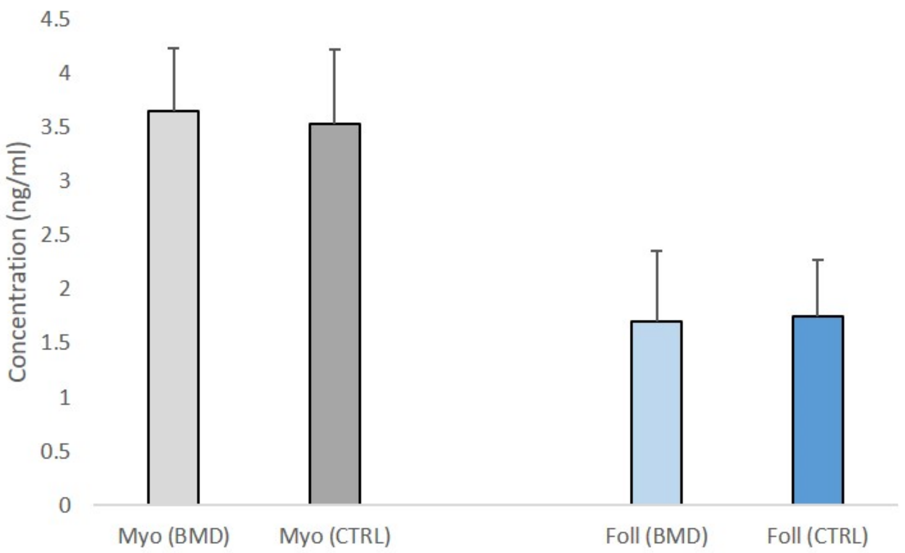

Figure S1 Myostatin (Myo) and follistatin (Foll) levels in plasma of eight Becker muscular dystrophy (BMD) and six controls. Data were presented as mean + standard deviation.

Table S1. TaqMan microRNA primer sequences.

MiRNA	Mature miRNA sequence	Assay ID
hsa-miR-1-3p	UGGAAUGUAAAGAAGUAUGUAU	002222
hsa-miR-206	UGGAAUGUAAGGAAGUGUGUGG	000510
hsa-miR-133a-3p	UUUGGUCCCCUUCAACCAGCUG	002246
hsa-miR-133b	UUUGGUCCCCUUCAACCAGCUA	002247
hsa-miR-155-5p	UUAAUGCUAAUCGUGAUAGGGGU	002623
hsa-miR-221	AGCUACAUUGUCUGCUGGGUUUC	000524
hsa-miR-146b	UGAGAACUGAAUUCCAUAGGCU	001097
cel-miR-39-3p	UCACCGGGUGUAAAUCAGCUUG	000200
hsa-miR-16	UAGCAGCACGUAAAUAUUGGCG	000391
U6 snRNA	GTGCTCGCTTCGGCAGCACATATACTAAAA TTGGAACGATACAGAGAAGATTAGCATGG CCCCTGCGCAAGGATGACACGCAAATTCGT GAAGCGTTCCATATTTT	001973

Table S2. Fatty infiltration with Mercuri score in lower limb muscles.

BMD Patients	Thigh Muscles		Leg Muscles	
	**Quadriceps**	**Hamstrings**	**Tibialis Anterior**	**Gastrocnemius**
**Patient 1**	3	4	2a	3
**Patient 2**	3	4	2a	4
**Patient 3**	0	0	0	0
**Patient 4**	1	1	0	1
**Patient 5**	N.D.	N.D.	N.D.	N.D.
**Patient 6**	0	0	0	0
**Patient 7**	1	2a	0	1
**Patient 8**	1	2a	0	2a

N.D.: not determined
